# Association of inflammatory markers with clinical outcomes in atrial fibrillation: a meta-analysis

**DOI:** 10.3389/fcvm.2025.1504163

**Published:** 2025-12-17

**Authors:** Xiaomei Chen, Xuge Zhang, Xiang Fang, Shenghong Feng

**Affiliations:** 1 Department of Cardiology, Dazhou Second People’s Hospital, Dazhou, Sichuan, China; 2 Department of Otorhinolaryngology Head and Neck Surgery, Dazhou Second People’s Hospital, Dazhou, Sichuan, China

**Keywords:** neutrophil-to-lymphocyte ratio, platelet-to-lymphocyte ratio, systemic immune-inflammation index, atrial fibrillation, meta-analysis

## Abstract

**Background:**

Inflammatory markers are increasingly recognized as key contributors to the pathogenesis and progression of atrial fibrillation (AF). This meta-analysis aims to systematically assess the prognostic significance of various lymphocyte-based inflammation indices, including the neutrophil-to-lymphocyte ratio (NLR), platelet-to-lymphocyte ratio (PLR), and systemic immune-inflammation index (SII) with clinical outcomes in AF.

**Methods:**

A comprehensive search was conducted in multiple databases until March 24, 2024. The included studies evaluated lymphocyte-based indices in relation to AF prognosis using a random-effects model. Weighted Mean Differences, Hazard ratios, and Odds Ratios with 95% Confidence Intervals were calculated. Subgroup and sensitivity analyses were performed, and evidence quality was assessed using the Grading of Recommendations Assessment, Development, and Evaluation (GRADE) framework.

**Results:**

Twenty-one studies involving 63,687 patients with AF were included. Higher NLR was associated with increased risks of all-cause mortality (HR: 1.50, 95% CI: 1.16–1.92; *I*² = 74%), stroke (HR: 1.42, 95% CI: 1.26−1.61; *I*² = 0%), AF recurrence (OR: 1.47, 95% CI: 1.17−1.86; *I*² = 93%), and left atrial thrombosis (OR: 2.12, 95% CI: 1.41−3.19; *I*² = 82%). Sensitivity analyses yielded similar estimates. Evidence for PLR and SII was limited to two studies each for left atrial thrombosis, with inconsistent results and high heterogeneity; therefore, no firm conclusions could be drawn. Exploratory subgroup analyses suggested lower heterogeneity in larger studies, but tests for subgroup differences were underpowered. Overall certainty of evidence ranged from low to very low by GRADE.

**Conclusion:**

Higher NLR shows an observational association with adverse outcomes in AF, but the certainty of evidence is low. Evidence for PLR and SII is extremely limited and inconsistent, precluding meaningful conclusions. Further large, well-designed prospective studies with standardized measurements are required.

**Systematic Review Registration:**

https://www.crd.york.ac.uk/PROSPERO/view/CRD42024540368, identifier CRD42024540368.

## 1 Introduction

Atrial fibrillation (AF), the most common cardiac arrhythmia globally, affects approximately 50 million individuals worldwide. As the global population ages, the prevalence of AF is expected to increase (1, 2). AF significantly elevates the risk of cardiovascular diseases and is closely associated with all-cause mortality, strokes, and severe cardiovascular complications (3). Despite advances in management, predicting clinical outcomes in AF remains a challenge, necessitating reliable biomarkers to guide individualized treatment strategies. Emerging evidence suggests that AF is not merely an electrical disorder but also involves structural and functional remodeling of the atria, often referred to as atrial cardiomyopathy (4). Inflammation is a key contributor to this process, promoting atrial fibrosis, endothelial dysfunction, and thrombogenicity, all of which increase the risk of AF-related adverse events (5). Therefore, inflammatory biomarkers may provide valuable prognostic insights into AF progression and associated complications.

Inflammation plays a crucial role in the development and recurrence of cardiovascular diseases (5, 6). Lymphocyte-based inflammatory index, including the neutrophil-to-lymphocyte ratio (NLR), platelet-to-lymphocyte ratio (PLR), systemic immune-inflammation index (SII), monocyte-to-lymphocyte ratio (MLR), and systemic inflammatory response index (SIRI), are readily available, cost-effective, and straightforward measures of inflammation. Research has shown that lymphocyte-based inflammatory indices correlate with poor outcomes in conditions such as acute heart failure (7, 8), acute coronary syndrome (9, 10), other coronary diseases (11, 12), and postoperative AF after cardiac surgery (13, 14). However, research on the relationship between these markers and adverse clinical outcomes like all-cause mortality, stroke, and AF recurrence following catheter ablation, as well as left atrial thrombosis among AF patients, remains limited. Therefore, investigating the predictive capabilities of lymphocyte-based inflammatory index for clinical outcomes in AF is essential.

This meta-analysis assessed the predictive value of lymphocyte-based inflammatory index for clinical outcomes in AF patients. By examining studies on all-cause mortality, AF recurrence post-catheter ablation, stroke, and left atrial thrombosis, this analysis provides a robust evidence base for evaluating the effectiveness of these indices as potential biomarkers in managing AF. Our research assesses the efficacy of these indices as prognostic biomarkers, which could be crucial for determining prognostic risks in AF patients and guiding targeted preventive treatments.

## 2 Methods

### 2.1 Protocol registration

This meta-analysis was conducted according to Preferred Reporting Items for Systematic Reviews and Meta-Analyses (PRISMA) 2020 guidelines (Supplementary Table S1) and was previously registered with PROSPERO (CRD42024540368).

### 2.2 Search strategy

The Cochrane Library, Embase, Scopus, Web of Science, and PubMed were searched from their respective inceptions until March 24, 2024, using the following strategy: “lymphocytes AND ratio AND (atrial fibrillation OR AF)”. The detailed search protocol is provided in the Supplementary Table S2.

The search strategy was developed by the authors in accordance with the PRISMA 2020 guidelines. Although a medical librarian was not directly consulted, the strategy was refined through multiple pilot runs and cross-checks with previously published meta-analyses in related fields to ensure comprehensiveness and accuracy. Reference lists of included studies and relevant reviews were also manually screened to identify additional eligible articles.

### 2.3 Inclusion and exclusion criteria

Studies that met the following criteria were included: 1) Examined the predictive value of lymphocyte-related inflammatory indices in AF patients; 2) Evaluated the correlation between lymphocyte-based inflammatory indices (NLR, PLR, MLR, SII, SIRI) and prognostic outcomes (all-cause mortality, stroke, post-catheter ablation AF recurrence, left atrial thrombosis); 3) Provided extractable data; for inclusion in the meta-analysis, at least two studies must have evaluated the same inflammatory index in relation to the same outcome measure to ensure data pooling feasibility; 4) Prioritized studies with the largest sample size and most recent data when multiple studies used the same patient cohort; 5) Published in English or Chinese, or translated into these languages.

The exclusion criteria were as follows: 1) reviews, meta-analysis, letters, case reports, and comments; 2) non-clinical studies; 3) studies that did not investigate the association between lymphocyte-related inflammatory indices (NLR, PLR, MLR, SII, SIRI) and clinical outcomes in atrial fibrillation.

### 2.4 Literature screening, data extraction

Two investigators (XMC, XGZ) independently screened articles. The two authors (XMC, XGZ) independently extracted general study information (authors, publication year, country) and baseline demographic and clinical characteristics (age, gender, NLR, PLR, SII, MLR, SIRI, all-cause mortality, stroke, AF recurrence, left atrial thrombus). The third author (SHF) verified the extracted data. All the disagreements were resolved through discussions among the third author (SHF).

### 2.5 Definitions and outcomes

The outcome indicators for AF included all-cause mortality, stroke, AF recurrence, and left atrial thrombosis. AF recurrence was categorized into early and late recurrence. Early recurrence of AF was defined as any atrial tachyarrhythmia (including AF, atrial flutter, and atrial tachycardia) continuously recorded for at least 30 s within a blanking period of three months. Late recurrence of AF was defined as the continuous recording of any 30-seconds atrial tachyarrhythmia (including AF, atrial flutter, and atrial tachycardia) occurring after the three-month blanking period.

### 2.6 Quality assessment

The quality of the included studies was evaluated using the Newcastle-Ottawa Quality Assessment Scale (NOS), with scores ranging from 0 to 9 (15). Studies with a NOS score of ≥7 were considered high-quality. Discrepancies arising during the evaluation process were resolved through consensus.

### 2.7 Data synthesis and analysis

Analyses were conducted in RevMan 5.4 and Stata 17.0 using inverse-variance weighting. For time-to-event outcomes we preferentially extracted hazard ratios (HRs); when HRs were unavailable, odds ratios (ORs) were used but not pooled together with HRs (separate syntheses or sensitivity analyses). Dichotomous outcomes were pooled as ORs with 95% CIs; continuous outcomes as (weighted) mean differences with 95% CIs. All meta-analyses used a random-effects model (DerSimonian–Laird).

Only contrasts with ≥2 independent studies and compatible effect measures were quantitatively pooled; contrasts not meeting these criteria were not synthesized or interpreted, and, if applicable, individual study estimates were tabulated in the Supplement for transparency. To avoid unit-of-analysis errors, when a study reported multiple cut-offs/categories for the same biomarker–outcome contrast, one effect per cohort was included according to a prespecified hierarchy [primary prespecified cut-off → ROC/Youden-derived cut-off → extreme-category contrast (highest vs. lowest)]; in all cases the most fully adjusted estimate was used. Alternative thresholds were examined in sensitivity analyses.

Heterogeneity was assessed with Cochran's Q and I² (substantial heterogeneity defined as *I*^2^ ≥ 50% or *P* ≤ 0.10). Prespecified exploratory subgroup analyses were performed by study design, region, sample size (e.g., ≥ 300 vs.<300), cut-off, measurement timing (pre- vs. post-procedure), and AF-recurrence timing (early vs. late); between-subgroup differences were tested using Q_between without multiplicity adjustment. Small-study effects were evaluated using funnel plots and Egger's regression only when *k* ≥ 5; formal tests were not performed when *k* < 5. Sensitivity analysis was conducted to evaluate result stability when the number of included studies was five or more. The Grading of Recommendations Assessment, Development, and Evaluation (GRADE) framework was used to evaluate the quality of evidence (16).

## 3 Results

### 3.1 Literature search and study characteristics

**Figure 1:**
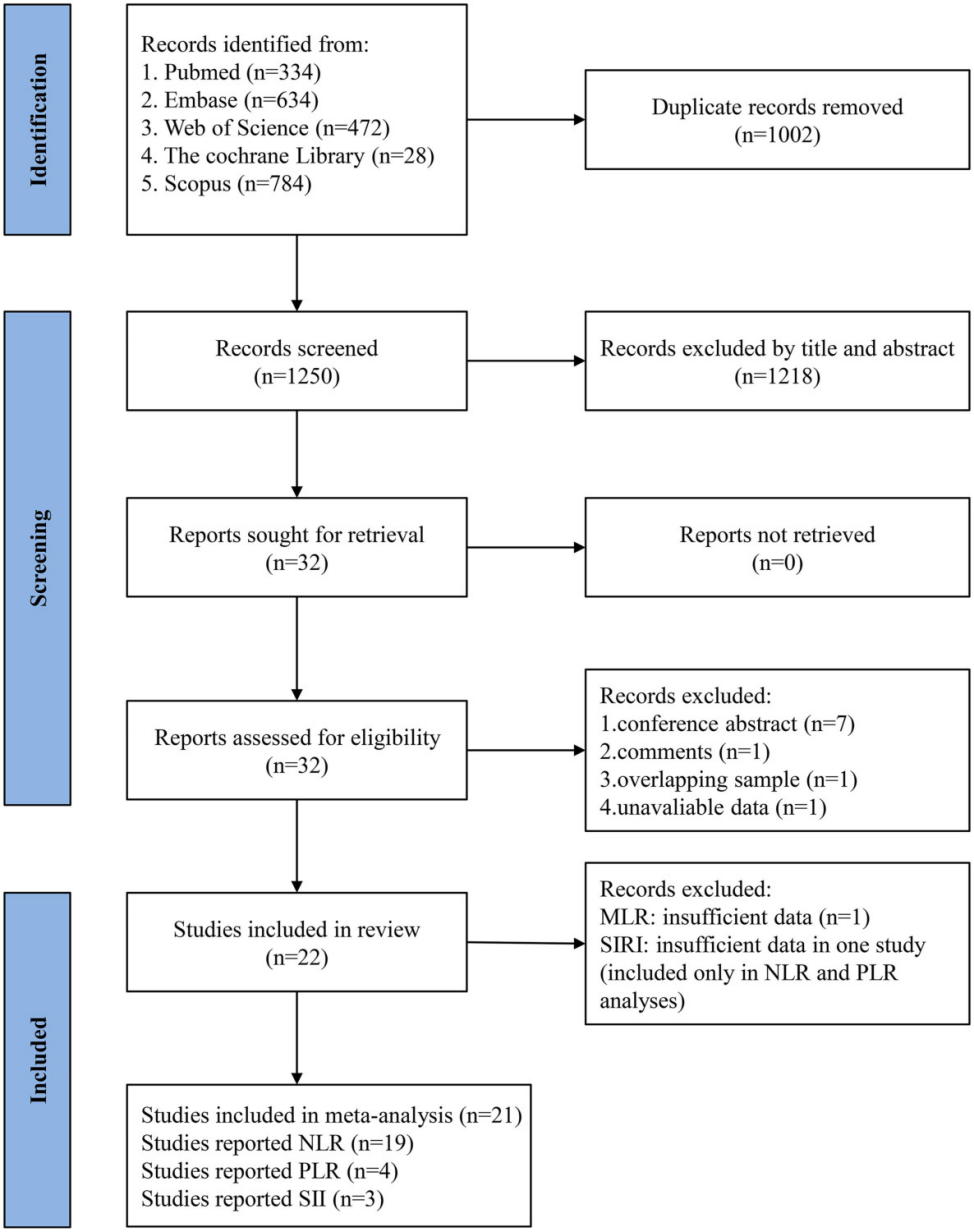
Flowchart of the systematic search and selection process.

Using the described search strategies, 2,252 articles were initially retrieved. Of these, 1,002 were excluded due to duplication. After screening titles and abstracts, 1,218 studies not meeting the specified inclusion and exclusion criteria were removed. Subsequently, the full texts of 32 studies were reviewed. During this phase, 7 conference abstracts, 1 commentary article, 1 study with overlapping populations, and 1 article lacking critical data were excluded. Additionally, two studies on MLR (17, 18) reported different outcomes, precluding meta-analysis, and only one study on SIRI (18) was available, which was insufficient for data pooling. Ultimately, 21 studies were included in the final analysis (18–38). Figure 1 presents the flow chart depicting the study selection process.

Table 1 presents the characteristics of the 21 included studies. Sample sizes ranged from 119 to 32,912, with a total of 63,687 participants. The reported age of participants ranged from 54.12 to 76.98 years. The studies comprised 18 retrospective and 3 prospective investigations. Regarding the inflammatory biomarkers, 19 studies focused exclusively on NLR, 4 on PLR, and 3 on SII. The research was conducted across multiple countries, including the United States, China, Japan, Korea, Ireland, Greece, and Turkey, with China as the primary research location. Details of the multivariable adjustment models and the covariates included in each study are summarized in Supplementary Table S3. According to the NOS, the studies were considered of high quality, with scores ranging from 7 to 8. Supplementary Table S4 provides details on NOS scores.

**Table 1:** Baseline characteristics of included studies.

Author	Year	Country	Study design	Follow up (months)	Time point	Sample size	Age	Male (%)	NLR cut-off	PLR cut-off	SII cut-off	Outcomes
Canpolat (25)	2013	Turkey	Prospective	19.0 ± 6.6	Pre	251	54.1 ± 10.9	52.2	3.15	/	/	AF recurrence (late recurrence)
Ertaş (28)	2013	Turkey	Retrospective	NA	Post	126	70.0 ± 10.2	41.3	3.17	/	/	stroke
Im (27)	2013	Korea	Retrospective	25.2 ± 14.5	Post	499	56.3 ± 11.4	68.9	5.6	/	/	AF recurrence (early recurrence)
Guo (26)	2014	China	Retrospective	30.5 ± 5.3	Post	379	49.7 ± 6.6	73.4	5.15	/	/	AF recurrence (late recurrence)
Saliba (29)	2015	Israel	Retrospective	12.0	Pre	32,912	75.0 ± 2.2	48.4	3	/	/	stroke
Yalcin (11)	2015	Turkey	Retrospective	NA	Pre	309	70.1 ± 9.8	47.0	2.59	/	/	left atrial thrombosis
Zhang (20)	2017	China	Retrospective	15.4 ± 3.5	Pre	119	49.6 ± 8.1	81.5	1.64	/	/	AF recurrence (early recurrence)
Fukuda (34)	2018	Japan	Retrospective	NA	Pre	183	64.0 ± 9.0	69.0	2.5	/	/	left atrial thrombosis
Li (22)	2018	China	Prospective	NA	Pre	140	54.8 ± 6.2	50.7	NA	/	/	left atrial thrombosis
Bazoukis (24)	2019	Greece	Retrospective	26.2 ± 12.1	Post	346	59.0 ± 11.0	65.0	3.9	/	/	AF recurrence (late recurrence)
He (21)	2021	China	Retrospective	NA	Pre	494	61.0 ± 10.0	61.7	2.22	/	/	left atrial thrombosis
Wu (30)	2021	China	Retrospective	39.8 ± 19.4	Pre	1,269	63.5 + 12.5	51.6	1.67	/	/	all-cause mortality
Etli (33)	2022	Turkey	Retrospective	NA	Pre	214	60.2 ± 14.8	59.8	NA	/	/	left atrial thrombosis
Tang (35)	2022	China	Retrospective	NA	Pre	207	61.9 ± 9.0	55.6	1.85	/	/	left atrial thrombosis
Xiang (23)	2022	China	Retrospective	NA	pre	178	68.8 ± 11.2	64.0	/	160.9	/	left atrial thrombosis
Deng (32)	2023	China	Retrospective	NA	Pre	569	62.1 ± 11.5	35.0	2.57	/	/	left atrial thrombosis
Dolu (37)	2023	Turkey	Retrospective	NA	Pre	403	60.8 ± 12.3	56.6	2.63	131.5	693.6	left atrial thrombosis
Fagundes (31)	2023	United States	Prospective	33.6	Pre and post	19,697	NA	NA	/	NA	/	all-cause mortality, stroke
Huang (19)	2023	China	Retrospective	15.1 ± 9.3	Pre	638	65.8 ± 10.5	48.0	/	NA	/	AF recurrence (late recurrence)
Zhou (18)	2023	China	Retrospective	NA	Pre	434	56.9 ± 9.1	36.2	2.663	/	423.334	left atrial thrombosis, all-cause mortality
Li (38)	2024	United States	Retrospective	1.0	Pre	4,562	76.0 ± 2.3	60.4	/	159.77	1,222.3	all-cause mortality

NLR, neutrophil to lymphocyte ratio; PLR, platelet to lymphocyte ratio; SII, systemic immune-inflammation index; MLR, monocyte to lymphocyte ratio; SIRI, systemic inflammation response index; AF, atrial fibrillation; NA, not available.

### 3.2 Predictive value of NLR

#### 3.2.1 All-cause mortality

**Figure 2:**
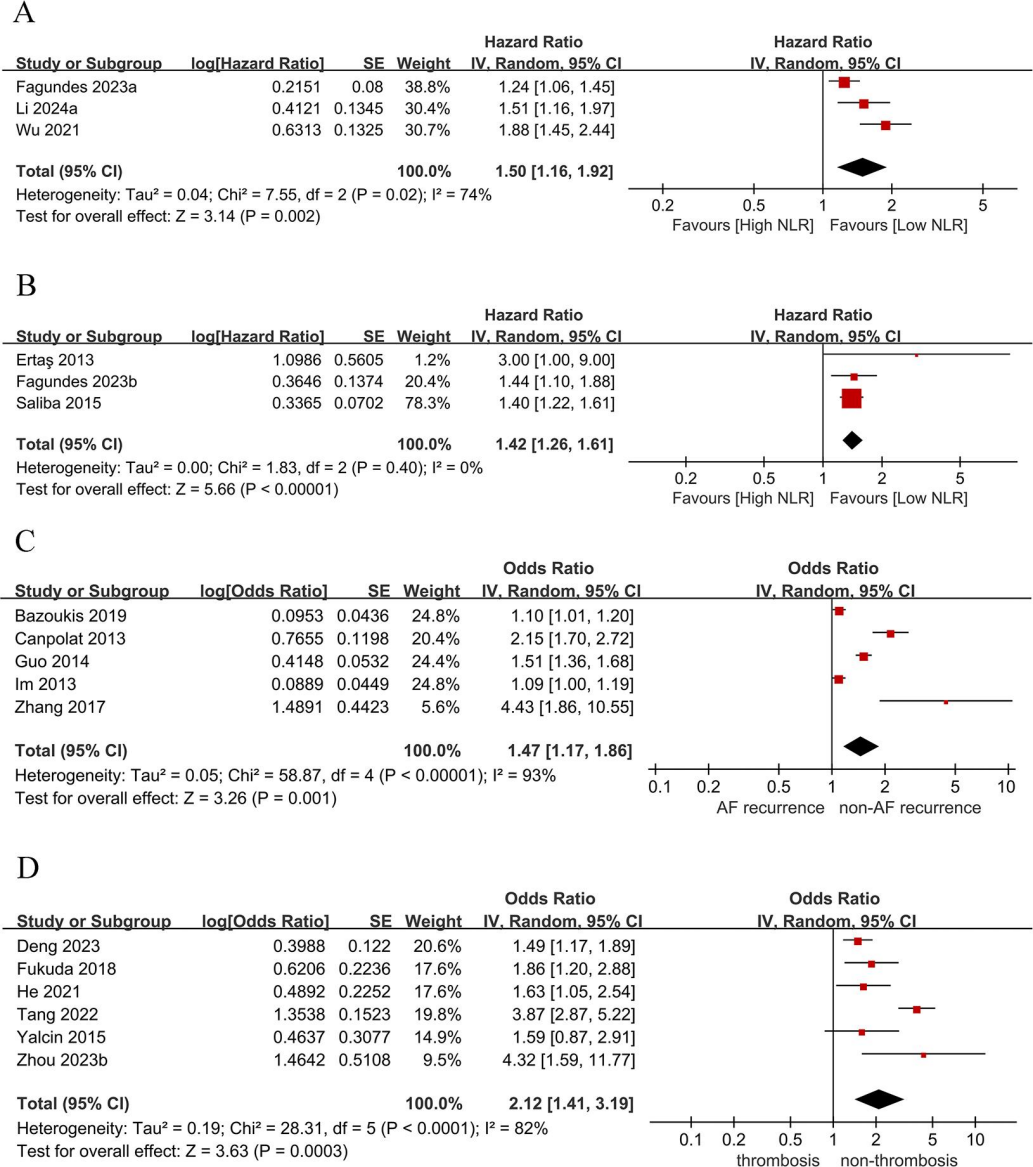
Forest plot of HRs and ORs showing associations between lymphocyte-based inflammatory markers and clinical outcomes in patients with AF: **(A)** NLR and All-cause mortality; **(B)** NLR and stroke; **(C)** NLR and AF recurrence; **(D)** NLR and left atrial thrombosis.

Three studies assessed the association between categorized NLR and all-cause mortality in AF patients. A higher NLR was associated with an increased risk of all-cause mortality (HR: 1.50, 95% CI: 1.16–1.92, *P* = 0.002, *I*^2^ = 74%) (Figure 2A).

#### 3.2.2 Stroke

Three studies assessed the association between categorized NLR and stroke in AF. A higher NLR was associated with an increased stroke risk (HR: 1.42, 95% CI: 1.26–1.61, *P* < 0.00001, *I*^2^ = 0%) (Figure 2B).

#### 3.2.3 AF recurrence

Five studies assessed the association between categorized NLR and AF recurrence. A higher NLR was associated with an increased AF recurrence risk (OR: 1.47, 95% CI: 1.17–1.86, *P* = 0.001, *I*^2^ = 93%) (Figure 2C).

#### 3.2.4 Left atrial thrombosis

Six studies assessed the association between categorized NLR and left atrial thrombosis. A higher NLR was associated with an increased left atrial thrombosis risk (OR: 2.12, 95% CI: 1.41–3.19, *P* = 0.0003, *I*^2^ = 82%) (Figure 2D).

### 3.3 Sensitivity analysis and publication bias assessment

Leave-one-out sensitivity analyses were performed for AF recurrence and left atrial thrombosis by sequentially omitting each study to assess its influence on the pooled ORs. The pooled estimates remained stable; no single study materially altered the magnitude or direction of the effect (Supplementary Figure S1).

Visual inspection of funnel plots suggested slight asymmetry (Supplementary Supplementary Figure S2). Consistent with our prespecified rule (*k* ≥ 5), Egger's regression did not indicate small-study effects for the categorized NLR analyses (AF recurrence: *P* = 0.146; left atrial thrombosis: *P* = 0.440). Given the small numbers of studies, these tests were underpowered, and results should be interpreted cautiously.

### 3.4 Subgroup analysis

Given substantial between-study heterogeneity in several contrasts, we performed prespecified exploratory subgroup analyses by study design, region, sample size (≥300 vs. <300), NLR cut-off (≥2.5 vs. <2.5), measurement timing (pre- vs. post-procedure), and AF-recurrence timing (early vs. late). Overall, higher NLR was associated with greater odds of left atrial thrombosis across subgroups; within-subgroup heterogeneity tended to be lower in larger studies. For AF recurrence, the magnitude of association appeared to differ by region and recurrence timing; however, several strata included ≤3 studies (some single-study), so formal tests for subgroup differences (Q_between) were underpowered, and findings should be interpreted cautiously. Detailed pooled effects (k, ORs with 95% CIs, and *I*^2^) are shown in Table 2.

**Table 2:** Subgroup analysis of the association between NLR and prognosis in patients with AF.

Subgroup	AF recurrence				Left atrial thrombosis			
	Study	OR [95%CI]	*P* value	*I* ^2^	Study	OR [95%CI]	*P* value	*I* ^2^
Total	5	1.47 [1.17–1.86]	0.001	93.00%	6	2.12 [1.41–3.19]	0.0003	82.00%
Study design								
Prospective	1	2.15 [1.70–2.73]	<0.0001	/	/	/	/	/
Retrospective	4	1.30 [1.05–1.62]	0.016	91.70%	/	/	/	/
Region								
Asia	3	1.48 [1.05–2.09]	0.024	93.20%	5	2.21 [1.44–3.40]	<0.0001	80.60%
Europe	2	1.52 [0.79–2.94]	0.209	96.30%	1	1.59 [0.73–3.49]	0.247	/
Sample size								
>300	3	1.22 [1.00–1.48]	0.049	92.70%	4	1.67 [1.25–2.21]	<0.0001	27.40%
<300	2	2.72[1.40–5.30]	0.003	59.90%	2	2.71 [1.44–3.11]	0.007	83.50%
Cut-off values								
***>2.5***	4	1.37 [0.10–1.72]	0.005	94.00%	3	1.80 [1.14–2.84]	0.01	51.00%
***≤2.5***	1	4.43 [1.86–10.55]	0.001	93.20%	3	2.32 [1.30–4.14]	0.005	85.00%
≥3	4	1.37 [0.10–1.72]	0.005	94.00%	/	/	/	/
<3	1	4.43[1.86–10.55]	0.001	93.20%	/	/	/	/
Time point								
Pre	2	2.72[1.40–5.30]	0.003	59.90%	/	/	/	/
Post	3	1.22 [1.00–1.48]	0.049	92.70%	/	/	/	/
AF recurrence								
Early recurrence	2	2.05[0.52–8.05]	0.301	93.20%	/	/	/	/
Late recurrence	3	1.50 [1.09–2.07]	0.012	95.00%	/	/	/	/

NLR, neutrophil to lymphocyte ratio; AF, Atrial fibrillation; OR, odds ratio; CI, confidence interval.

### 3.5 Predictive value of PLR

#### 3.5.1 Left atrial thrombosis

**Figure 3:**
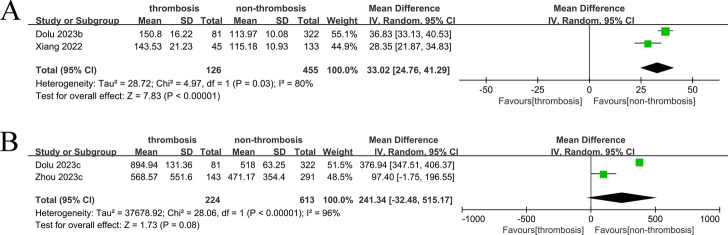
Forest plot of WMDs showing the association between left atrial thrombosis risk and PLR/SII: **(A)** PLR and left atrial thrombosis; **(B)** SII and left atrial thrombosis.

Two studies reported on continuous PLR data related to left atrial thrombosis. The PLR was significantly higher in the left atrial thrombus group compared to the non-thrombus group (WMD: 33.02, 95% CI: 24.76–41.29, *P* < 0.00001, *I*^2^ = 80%) (Figure 3A).

### 3.6 Predictive value of SII

#### 3.6.1 Left atrial thrombosis

Two studies reported on continuous SII data related to left atrial thrombosis. The SII showed no statistically significant difference between the left atrial thrombus group and the non-thrombus group (WMD: 241.34, 95% CI: −32.48–515.17, *P* = 0.08, *I*^2^ = 96%) (Figure 3B).

### 3.7 GRADE classification

The GRADE framework was employed to assess the quality of evidence and strength of recommendations. Within this framework, the stroke outcome indicator related to NLR was rated as low quality. Despite the absence of significant downgrading factors in the analyzed domains, the inherent low level of evidence in observational study designs, specifically cohort and case-control studies, maintained this classification. For other outcome measures, the evidence quality was further rated as very low due to significant risk of bias identified in at least one critical area, such as study design, consistency of results, or measurement precision. Table 3 provides detailed scores for these assessments.

**Table 3:** GRADE evidence profile table.

Outcomes	Markers	No. of studies	Prospective	Retrospective	Risk of bias	Inconsistency	Indirectness	Imprecision	Publication bias	Plausible confounding	Magnitude of effect	Dose-response gradient	Quality
All-cause mortality	NLR (HR)	3	1	2	No serious risk	Serious	No serious	No Serious	Undetected	Would not reduce effect	No	No	Very low
						Inconsistency	Indirectness	Imprecision					
Stroke	NLR (HR)	3	0	3	No serious risk	no serious	No serious	No Serious	Undetected	Would not reduce effect	No	No	low
						Inconsistency	Indirectness	Imprecision					
AF recurrence	NLR (OR)	5	1	4	No serious risk	Serious	No serious	Serious	Undetected	Would reduce effect	No	No	Very low
						inconsistency	Indirectness	Imprecision					
Left atrial thrombosis	NLR (OR)	6	0	6	Am	Serious	No serious	Serious	Undetected	Would not reduce effect	Yes	No	Very low
						Inconsistency	Indirectness	Imprecision					
Left atrial thrombosis	PLR (WMD)	2	0	2	No serious risk	Serious	No serious	Serious	Undetected	Would not reduce effect	No	No	Very low
						Inconsistency	Indirectness	Imprecision					
Left atrial thrombosis	SII (WMD)	2	0	2	No serious risk	Serious	No serious	Serious	Undetected	Would not reduce effect	No	No	Very low
						Inconsistency	Indirectness	Imprecision					

GRADE, Grading of Recommendations Assessment, Development, and Evaluation; NLR, neutrophil to lymphocyte ratio; PLR, platelet to lymphocyte ratio; SII, systemic immune-inflammation index; AF, Atrial fibrillation; HR, hazard ratio; OR, odds ratio; CI, confidence interval; WMD, weighted mean difference.

## 4 Discussion

### 4.1 Key findings

This meta-analysis of 21 studies (*n* = 63,687) shows that higher NLR is associated with increased risks of all-cause mortality, stroke, AF recurrence, and left atrial thrombosis, with pooled estimates generally ranging from 1.4 to 2.1. Substantial heterogeneity was observed across several contrasts, although sensitivity analyses supported the stability of the pooled results and no conclusive small-study effects were detected. Exploratory subgroup analyses indicated that heterogeneity was partly related to sample size and regional differences, but statistical power within subgroups was limited. Evidence for PLR and SII was very limited, with only two studies available for each and marked heterogeneity; these analyses are therefore exploratory and not suitable for firm prognostic inference. The certainty of evidence for the NLR–outcome associations was rated as low to very low according to GRADE.

### 4.2 Potential mechanisms linking inflammation to AF outcomes

AF is increasingly recognized as an inflammatory-driven arrhythmia, where systemic inflammation contributes to its initiation, progression, and thromboembolic complications. In this context, lymphocyte-based inflammatory markers such as NLR, PLR, and SII may serve as valuable indicators reflecting underlying pathophysiological mechanisms.

Atrial cardiomyopathy plays a central role in AF onset, maintenance, and thromboembolic complications. It is characterized by structural abnormalities (atrial fibrosis, dilation), electrical remodeling (conduction disturbances), and autonomic dysregulation (39, 40). Inflammation is a key driver of atrial cardiomyopathy, where chronic inflammatory states—mediated by cytokines such as IL-6, TNF-α, and CRP—induce atrial fibrosis, alter intercellular connexins, and promote AF persistence (41–43). Additionally, endothelial dysfunction and platelet activation, both influenced by systemic inflammation, may contribute to the heightened stroke risk observed in AF patients (44). NLR has been proposed as a surrogate for inflammatory burden in AF, with elevated levels correlating with the extent of atrial fibrosis and serving as a predictor of AF recurrence and left atrial thrombus formation (45). These findings underscore the intricate link between inflammation and atrial remodeling, suggesting that inflammatory markers may provide insights into AF pathophysiology and risk stratification.

Catheter ablation, including radiofrequency catheter ablation (RFCA) and pulsed field ablation (PFA), is an effective treatment for symptomatic AF. RFCA utilizes thermal energy to disrupt abnormal conduction pathways, whereas PFA selectively ablates myocardial tissue using electroporation while sparing adjacent structures such as the esophagus and phrenic nerve. However, inflammation plays a crucial role in post-ablation AF recurrence. RFCA has been associated with a significant elevation in inflammatory mediators (IL-6, TNF-α, CRP) post-procedure, which may contribute to early AF recurrence (ERAF) and potentially impact long-term outcomes (46–49). In contrast, PFA appears to elicit a milder inflammatory response, with lower postoperative NLR and CRP levels, which may be linked to a reduced risk of AF recurrence (50). Recent evidence further supports that higher postoperative NLR is associated with increased rates of ERAF, suggesting a role for inflammation in post-ablation arrhythmogenesis (51). Similarly, elevated PLR and SII have been correlated with higher AF recurrence rates and left atrial thrombus formation following ablation (46). These findings highlight the importance of inflammation in procedural outcomes, reinforcing the need to consider inflammatory markers in post-ablation risk stratification. These modality-specific differences in inflammatory activation may partly explain the variation in early AF recurrence observed following RFCA vs. PFA. Moreover, the association between higher postoperative NLR and increased ERAF risk suggests that NLR may capture procedure-related inflammatory injury, linking biomarker dynamics to ablation-specific prognostic differences.

Together, these mechanistic insights suggest that inflammation is a crucial modulator of AF pathogenesis, influencing both disease progression and treatment response. Future studies should explore targeted anti-inflammatory strategies to mitigate AF burden and improve procedural outcomes.

In addition to these biological mechanisms, several important clinical and methodological confounders may also have contributed to the variability of the observed associations. First, AF duration and AF type (paroxysmal vs. persistent), both of which strongly influence baseline inflammatory burden and AF prognosis, were inconsistently reported and rarely adjusted across included studies. Second, comorbidities such as heart failure, hypertension, diabetes, and renal dysfunction can independently elevate inflammatory markers and modify AF outcomes, yet the degree of adjustment varied substantially among studies. Third, anticoagulation status and antiarrhythmic drug use, which profoundly influence stroke and thrombus risk, were not uniformly accounted for. Treatment modality represents another key confounder, particularly in analyses involving catheter ablation: radiofrequency ablation and pulsed field ablation elicit markedly different inflammatory responses, potentially influencing postoperative AF recurrence. Together, these discrepancies in baseline characteristics, treatment strategies, and adjustment models likely contributed to the substantial heterogeneity observed, beyond statistical explanations alone. Future studies should adopt standardized covariate adjustment frameworks to better isolate the independent prognostic value of inflammatory indices.

### 4.3 Clinical implications and incremental predictive value

The clinical implications of these findings should be interpreted cautiously. Whether NLR provides prognostic value beyond established risk scores such as CHA₂DS₂-VASc is unknown, as few included studies adjusted for key clinical predictors and none evaluated incremental metrics (e.g., NRI, IDI). Evidence supporting combined use of NLR with traditional scores in AF is therefore lacking. No existing studies have rigorously examined combined NLR–risk score models in AF populations, underscoring a current evidence gap. Differences in inflammatory response between RFCA and PFA also suggest potential modality-specific trajectories, but this remains unproven. Overall, NLR should be regarded as a research biomarker until studies establish standardized measurement, validated cutoffs, and demonstrable incremental predictive value.

### 4.4 Comparison with previous studies

Compared with previous studies, our meta-analysis provides a more comprehensive evaluation of multiple inflammatory markers beyond NLR, encompassing PLR, and SII in relation to various clinical outcomes in AF. The findings align with and expand upon those of Lu et al. (52), who demonstrated a significant association between NLR and stroke risk in AF patients, but our study incorporates a broader scope of inflammatory markers and additional prognostic outcomes, including all-cause mortality, AF recurrence, and left atrial thrombosis. Similarly, Lekkala et al. (53) reported the relationship between NLR and AF recurrence in patients undergoing catheter ablation, but their analysis lacked assessments of other inflammatory markers and did not apply subgroup analysis or the GRADE framework. In contrast, our study addresses these gaps by systematically evaluating the quality of evidence and identifying potential sources of heterogeneity. Furthermore, prior meta-analyses by Dentali et al. (54) and Vakhshoori et al. (55) have established the prognostic role of NLR in other cardiovascular conditions, including acute coronary syndrome and heart failure, findings that our study corroborates in the context of AF. The prognostic utility of SII, which Zhang et al. (56) linked to major adverse cardiac events in post-PCI patients, was also reinforced by our findings, highlighting SII as an important inflammatory marker associated with AF-related complications. By integrating these insights, our study provides an updated and more expansive perspective on the role of inflammation in AF, suggesting that lymphocyte-based inflammatory markers may serve as valuable prognostic indicators across a spectrum of cardiovascular diseases.

### 4.5 Limitations

This study has several limitations. First, most included studies were retrospective, and variations in NLR, PLR, and SII cutoff values introduced heterogeneity, affecting interpretability. Despite our standardized approach to extracting HR/OR, differences in NLR stratification may have influenced pooled estimates. Future studies should establish uniform classification criteria or report continuous values for better comparability.

Second, follow-up durations varied, particularly in all-cause mortality analysis. One study had a 30-day follow-up, while others exceeded 12 months, complicating risk estimation. Inflammatory markers may reflect transient stress acutely but indicate chronic remodeling long-term. Standardized follow-up periods are needed to clarify these temporal dynamics.

Third, substantial heterogeneity was observed, likely due to differences in population characteristics, biomarker assessments, and study designs. Despite subgroup and sensitivity analyses, residual bias cannot be excluded. A key source of heterogeneity was the lack of standardized NLR cutoffs, which were derived using diverse statistical approaches and applied to different blood sampling time points across studies. Moreover, true harmonization of NLR values was not feasible because individual patient–level continuous data were unavailable, making recalculation or reclassification impossible within a study-level meta-analytic framework. This methodological variability not only constrained comparability across studies but also limits the practical clinical applicability of NLR, as no validated or universally accepted threshold currently exists. Future research should establish consensus definitions, standardized measurement protocols, and clinically validated cutoffs—ideally through large, prospective cohorts—before NLR can be reliably incorporated into routine risk stratification algorithms. Additionally, variations in adjusted covariates contributed to heterogeneity. Although we prioritized HRs/ORs from adjusted models, the included variables differed (e.g., cardiovascular risk factors vs. inflammatory markers), affecting comparability. To enhance the robustness of future meta-analyses, standardized adjustment strategies should be implemented. Furthermore, several clinically important determinants of both inflammatory marker levels and AF prognosis—such as AF type (paroxysmal vs. persistent), AF duration, major comorbidities (e.g., heart failure, hypertension, diabetes, renal dysfunction), and the use of anticoagulants or antiarrhythmic drugs—were inconsistently reported and rarely adjusted for across studies. In analyses involving catheter ablation, differences in procedural modality (radiofrequency vs. pulsed field ablation), which are known to elicit distinct inflammatory responses, may also have contributed to between-study variability. The inability to account adequately for these clinical and treatment-related confounders further limits interpretation of the pooled associations and likely explains part of the residual heterogeneity.

Finally, the limited number of studies restricted a comprehensive synthesis of individual inflammatory markers. While we removed biomarkers reported in only one study (MLR and SIRI), we retained those with two studies, including PLR and SII, as they met the minimum threshold for meta-analysis. However, results from these markers should be interpreted with caution due to the small sample size, substantial heterogeneity, and potential publication bias. The limited number of studies increases the risk of overestimated effect sizes, and variability in biomarker cutoff values further complicates comparability. Given these limitations, the PLR and SII findings should be regarded as exploratory only, as the very small evidence base and inconsistent effect directions preclude any reliable prognostic inference. Future large-scale prospective studies with standardized methodologies and predefined biomarker thresholds are needed to validate their clinical relevance and refine risk stratification.

## 5 Conclusion

This meta-analysis indicates a possible association between higher NLR and adverse outcomes in atrial fibrillation, including all-cause mortality, stroke, AF recurrence, and left atrial thrombosis; however, these findings are limited by substantial heterogeneity and an overall low certainty of evidence. Evidence for PLR and SII was extremely limited and heterogeneous, and current data do not support any reliable prognostic or clinically interpretable conclusions for these markers. Given these limitations, NLR should be interpreted cautiously as a potential signal rather than a validated risk stratification tool. Well-designed prospective studies with standardized measurement, uniform cut-offs, and comprehensive adjustment are required to clarify the independent prognostic value of lymphocyte-based inflammatory indices.

## Data Availability

The original contributions presented in the study are included in the article/Supplementary Material, further inquiries can be directed to the corresponding author/s.
