# Supplementary material for: Association of inflammatory markers with clinical outcomes in atrial fibrillation: a meta-analysis
Source: Front Cardiovasc Med. 2025 Dec 17;12:1504163. doi: 10.3389/fcvm.2025.1504163 (PMC12753891; doi:10.3389/fcvm.2025.1504163)
Supplement: Supplementary file 1 [file Datasheet1.docx]

Supplementary Materials

**Table S1.** PRISMA Checklist.

**Table S2.** Literature Search Strategy.

**Table S3**. Summary of Adjustment Models and Main Covariates Across Included Studies.

**Table S4.** (A) The Newcastle-Ottawa Quality Assessment Scale Score for Cohort Studies; (B) The Newcastle-Ottawa Quality Assessment Scale Score for Case-Control Studies.

**Figure S1.** Sensitivity Analysis of ORs for Associations Between NLR and Different Clinical Outcomes in Patients with AF.

**Figure S2.** Funnel Plots of ORs for Associations Between NLR and Different Clinical Outcomes in Patients with AF.

**Table S1.** PRISMA Checklist.

| **Section and Topic** | **Item #** | **Checklist item** | **Location where item is reported** |
| --- | --- | --- | --- |
| **TITLE** | | |  |
| Title | 1 | Identify the report as a systematic review. | Title |
| **ABSTRACT** | | |  |
| Abstract | 2 | See the PRISMA 2020 for Abstracts checklist. | Abstract |
| **INTRODUCTION** | | |  |
| Rationale | 3 | Describe the rationale for the review in the context of existing knowledge. | Introduction |
| Objectives | 4 | Provide an explicit statement of the objective(s) or question(s) the review addresses. | Introduction |
| **METHODS** | | |  |
| Eligibility criteria | 5 | Specify the inclusion and exclusion criteria for the review and how studies were grouped for the syntheses. | Methods |
| Information sources | 6 | Specify all databases, registers, websites, organisations, reference lists and other sources searched or consulted to identify studies. Specify the date when each source was last searched or consulted. | Methods |
| Search strategy | 7 | Present the full search strategies for all databases, registers and websites, including any filters and limits used. | Methods |
| Selection process | 8 | Specify the methods used to decide whether a study met the inclusion criteria of the review, including how many reviewers screened each record and each report retrieved, whether they worked independently, and if applicable, details of automation tools used in the process. | Methods |
| Data collection process | 9 | Specify the methods used to collect data from reports, including how many reviewers collected data from each report, whether they worked independently, any processes for obtaining or confirming data from study investigators, and if applicable, details of automation tools used in the process. | Methods |
| Data items | 10a | List and define all outcomes for which data were sought. Specify whether all results that were compatible with each outcome domain in each study were sought (e.g. for all measures, time points, analyses), and if not, the methods used to decide which results to collect. | Methods |
|  | 10b | List and define all other variables for which data were sought (e.g. participant and intervention characteristics, funding sources). Describe any assumptions made about any missing or unclear information. | Methods |
| Study risk of bias assessment | 11 | Specify the methods used to assess risk of bias in the included studies, including details of the tool(s) used, how many reviewers assessed each study and whether they worked independently, and if applicable, details of automation tools used in the process. | Methods |
| Effect measures | 12 | Specify for each outcome the effect measure(s) (e.g. risk ratio, mean difference) used in the synthesis or presentation of results. | Methods |
| Synthesis methods | 13a | Describe the processes used to decide which studies were eligible for each synthesis (e.g. tabulating the study intervention characteristics and comparing against the planned groups for each synthesis (item #5)). | Methods |
|  | 13b | Describe any methods required to prepare the data for presentation or synthesis, such as handling of missing summary statistics, or data conversions. | Methods |
|  | 13c | Describe any methods used to tabulate or visually display results of individual studies and syntheses. | Methods |
|  | 13d | Describe any methods used to synthesize results and provide a rationale for the choice(s). If meta-analysis was performed, describe the model(s), method(s) to identify the presence and extent of statistical heterogeneity, and software package(s) used. | Methods |
|  | 13e | Describe any methods used to explore possible causes of heterogeneity among study results (e.g. subgroup analysis, meta-regression). | Methods |
|  | 13f | Describe any sensitivity analyses conducted to assess robustness of the synthesized results. | Methods |
| Reporting bias assessment | 14 | Describe any methods used to assess risk of bias due to missing results in a synthesis (arising from reporting biases). | Methods |
| Certainty assessment | 15 | Describe any methods used to assess certainty (or confidence) in the body of evidence for an outcome. | Methods |
| **RESULTS** | | |  |
| Study selection | 16a | Describe the results of the search and selection process, from the number of records identified in the search to the number of studies included in the review, ideally using a flow diagram. | Results |
|  | 16b | Cite studies that might appear to meet the inclusion criteria, but which were excluded, and explain why they were excluded. | Results |
| Study characteristics | 17 | Cite each included study and present its characteristics. | Results |
| Risk of bias in studies | 18 | Present assessments of risk of bias for each included study. | Results |
| Results of individual studies | 19 | For all outcomes, present, for each study: (a) summary statistics for each group (where appropriate) and (b) an effect estimate and its precision (e.g. confidence/credible interval), ideally using structured tables or plots. | Results |
| Results of syntheses | 20a | For each synthesis, briefly summarise the characteristics and risk of bias among contributing studies. | Results |
|  | 20b | Present results of all statistical syntheses conducted. If meta-analysis was done, present for each the summary estimate and its precision (e.g. confidence/credible interval) and measures of statistical heterogeneity. If comparing groups, describe the direction of the effect. | Results |
|  | 20c | Present results of all investigations of possible causes of heterogeneity among study results. | Results |
|  | 20d | Present results of all sensitivity analyses conducted to assess the robustness of the synthesized results. | Results |
| Reporting biases | 21 | Present assessments of risk of bias due to missing results (arising from reporting biases) for each synthesis assessed. | Results |
| Certainty of evidence | 22 | Present assessments of certainty (or confidence) in the body of evidence for each outcome assessed. | Results |
| **DISCUSSION** | | |  |
| Discussion | 23a | Provide a general interpretation of the results in the context of other evidence. | Discussion |
|  | 23b | Discuss any limitations of the evidence included in the review. | Discussion |
|  | 23c | Discuss any limitations of the review processes used. | Discussion |
|  | 23d | Discuss implications of the results for practice, policy, and future research. | Discussion |
| **OTHER INFORMATION** | | |  |
| Registration and protocol | 24a | Provide registration information for the review, including register name and registration number, or state that the review was not registered. | Methods |
|  | 24b | Indicate where the review protocol can be accessed, or state that a protocol was not prepared. | Methods |
|  | 24c | Describe and explain any amendments to information provided at registration or in the protocol. | Methods |
| Support | 25 | Describe sources of financial or non-financial support for the review, and the role of the funders or sponsors in the review. | Declarations |
| Competing interests | 26 | Declare any competing interests of review authors. | Declarations |
| Availability of data, code and other materials | 27 | Report which of the following are publicly available and where they can be found: template data collection forms; data extracted from included studies; data used for all analyses; analytic code; any other materials used in the review. | Declarations |

*From:*  Page MJ, McKenzie JE, Bossuyt PM, Boutron I, Hoffmann TC, Mulrow CD, et al. The PRISMA 2020 statement: an updated guideline for reporting systematic reviews. BMJ 2021;372:n71. doi: 10.1136/bmj.n71

For more information, visit: <http://www.prisma-statement.org/>

**Table S2.** Literature Search Strategy.

**1.Pubmed**

| Search number | Query |
| --- | --- |
| #1 | Lymphocytes [MeSH Terms] |
| #2 | (((((Lymphocytes) OR (Lymphocyte)) OR (Lymphoid Cells)) OR (Cell, Lymphoid)) OR (Cells, Lymphoid)) OR (Lymphoid Cell) |
| #3 | Ratio |
| #4 | Atrial Fibrillation [MeSH Terms] |
| #5 | (((((((((((((((((((((((((Atrial Fibrillation) OR (Atrial Fibrillations)) OR (Fibrillation, Atrial)) OR (Fibrillations, Atrial)) OR (Auricular Fibrillation)) OR (Auricular Fibrillations)) OR (Fibrillation, Auricular)) OR (Fibrillations, Auricular)) OR (Persistent Atrial Fibrillation)) OR (Atrial Fibrillation, Persistent)) OR (Fibrillation, Persistent Atrial)) OR (Fibrillations, Persistent Atrial)) OR (Persistent Atrial Fibrillations)) OR (Familial Atrial Fibrillation)) OR (Atrial Fibrillation, Familial)) OR (Atrial Fibrillations, Familial)) OR (Familial Atrial Fibrillations)) OR (Fibrillation, Familial Atrial)) OR (Fibrillations, Familial Atrial)) OR (Paroxysmal Atrial Fibrillation)) OR (Atrial Fibrillation, Paroxysmal)) OR (Atrial Fibrillations, Paroxysmal)) OR (Fibrillation, Paroxysmal Atrial)) OR (Fibrillations, Paroxysmal Atrial)) OR (Paroxysmal Atrial Fibrillations)) OR (AF) |
| #6 | #1 OR #2 |
| #7 | #4 OR #5 |
| #8 | #6 AND #3 #7 |

**2.the Cochrane library**

| Search number | Query |
| --- | --- |
| #1 | MeSH descriptor: [Atrial Fibrillation] explode all trees |
| #2 | (Atrial Fibrillation, Paroxysmal):ti,ab,kw OR( Paroxysmal Atrial Fibrillations):ti,ab,kw OR( Atrial Fibrillations, Paroxysmal):ti,ab,kw OR( Paroxysmal Atrial Fibrillation):ti,ab,kw OR( Fibrillation, Paroxysmal Atrial):ti,ab,kw OR( Fibrillations, Paroxysmal Atrial):ti,ab,kw OR( Atrial Fibrillation, Persistent):ti,ab,kw OR( Fibrillation, Persistent Atrial):ti,ab,kw OR( Atrial Fibrillations, Persistent):ti,ab,kw OR( Fibrillations, Persistent Atrial):ti,ab,kw OR( Persistent Atrial Fibrillation):ti,ab,kw OR( Persistent Atrial Fibrillations):ti,ab,kw OR( Fibrillations, Atrial):ti,ab,kw OR( Fibrillation, Auricular):ti,ab,kw OR( Fibrillation, Atrial):ti,ab,kw OR( Atrial Fibrillations):ti,ab,kw OR( Fibrillations, Auricular):ti,ab,kw OR( Auricular Fibrillation):ti,ab,kw OR( Auricular Fibrillations):ti,ab,kw OR( Familial Atrial Fibrillations):ti,ab,kw OR( Fibrillation, Familial Atrial):ti,ab,kw OR( Atrial Fibrillations, Familial):ti,ab,kw OR( Fibrillations, Familial Atrial):ti,ab,kw OR( Familial Atrial Fibrillation):ti,ab,kw OR( Atrial Fibrillation, Familial):ti,ab,kw |
| #3 | MeSH descriptor: [Lymphocytes] explode all trees |
| #4 | (Cells, Lymphoid):ti,ab,kw OR (Lymphoid Cells):ti,ab,kw OR (Lymphoid Cell):ti,ab,kw OR (Cell, Lymphoid):ti,ab,kw OR (Lymphocyte):ti,ab,kw |
| #5 | (ratio) (Word variations have been searched) |
| #6 | #1 OR #2 |
| #7 | #3 OR #4 |
| #8 | #7 AND #5 AND #6 |

**3.Embase**

| Search number | Query |
| --- | --- |
| #1 | 'atrial fibrillation'/exp |
| #2 | 'atrium fibrillation':ab,ti OR 'auricular fibrilation':ab,ti OR 'auricular fibrillation':ab,ti OR 'cardiac atrial fibrillation':ab,ti OR 'cardiac atrium fibrillation':ab,ti OR 'fibrillation, heart atrium':ab,ti OR 'heart atrial fibrillation':ab,ti OR 'heart atrium fibrillation':ab,ti OR 'heart fibrillation atrium':ab,ti OR 'non-valvular atrial fibrillation':ab,ti OR 'nonvalvular atrial fibrillation':ab,ti OR 'atrial fibrillation':ab,ti |
| #3 | 'lymphocyte'/exp |
| #4 | 'blood lymphocyte':ab,ti OR 'f1 lymphocyte':ab,ti OR 'immune competent cell':ab,ti OR 'immune lymphocyte':ab,ti OR 'immune lymphoid cell':ab,ti OR 'immunocyte':ab,ti OR 'large lymphocyte':ab,ti OR 'lymph cell':ab,ti OR 'lymphocyte f1':ab,ti OR 'lymphocyte kinetics':ab,ti OR 'lymphocyte, immune':ab,ti OR 'lymphocytes':ab,ti OR 'memory lymphocyte':ab,ti OR 'small lymphocyte':ab,ti OR 'lymphocyte':ab,ti |
| #5 | ratio |
| #6 | #1 OR #2 |
| #7 | #3 OR #4 |
| #8 | #6 AND #5 AND #7 |

**4.Web of science**

| Search number | Query |
| --- | --- |
| #1 | ((((((((((((((((((((((((((TS=(Atrial Fibrillation)) OR TS=(Atrial Fibrillations)) OR TS=(Fibrillation, Atrial)) OR TS=(Fibrillations, Atrial)) OR TS=(Auricular Fibrillation)) OR TS=(Auricular Fibrillations)) OR TS=(Fibrillation, Auricular)) OR TS=(Fibrillations, Auricular)) OR TS=(Persistent Atrial Fibrillation)) OR TS=(Atrial Fibrillation, Persistent)) OR TS=(Atrial Fibrillations, Persistent)) OR TS=(Fibrillation, Persistent Atrial)) OR TS=(Fibrillations, Persistent Atrial)) OR TS=(Persistent Atrial Fibrillations)) OR TS=(Familial Atrial Fibrillation)) OR TS=(Atrial Fibrillation, Familial)) OR TS=(Atrial Fibrillations, Familial)) OR TS=(Familial Atrial Fibrillations)) OR TS=(Fibrillation, Familial Atrial)) OR TS=(Fibrillations, Familial Atrial)) OR TS=(Paroxysmal Atrial Fibrillation)) OR TS=(Atrial Fibrillation, Paroxysmal)) OR TS=(Atrial Fibrillations, Paroxysmal)) OR TS=(Fibrillation, Paroxysmal Atrial)) OR TS=(Fibrillations, Paroxysmal Atrial)) OR TS=(Paroxysmal Atrial Fibrillations)) OR TS=(AF) and Preprint Citation Index (Exclude – Database) |
| #2 | (((((TS=(Lymphocytes)) OR TS=(Lymphocyte)) OR TS=(Lymphoid Cells)) OR TS=(Cell, Lymphoid)) OR TS=(Cells, Lymphoid)) OR TS=(Lymphoid Cell) and Preprint Citation Index (Exclude – Database) |
| #3 | TS=(ratio) and Preprint Citation Index (Exclude – Database) |
| #4 | #3 AND #2 AND #1 and Preprint Citation Index (Exclude – Database) |

**5.Scopus**

| Search number | Query |
| --- | --- |
| #1 | TITLE-ABS-KEY ( "atrial fibrillation, paroxysmal" OR " paroxysmal atrial fibrillations" OR " atrial fibrillations, paroxysmal" OR " paroxysmal atrial fibrillation" OR " fibrillation, paroxysmal atrial" OR " fibrillations, paroxysmal atrial" OR " atrial fibrillation, persistent" OR " fibrillation, persistent atrial" OR " atrial fibrillations, persistent" OR " fibrillations, persistent atrial" OR " persistent atrial fibrillation" OR " persistent atrial fibrillations" OR " fibrillations, atrial" OR " fibrillation, auricular" OR " fibrillation, atrial" OR " atrial fibrillations" OR " fibrillations, auricular" OR " auricular fibrillation" OR " auricular fibrillations" OR " familial atrial fibrillations" OR " fibrillation, familial atrial" OR " atrial fibrillations, familial" OR " fibrillations, familial atrial" OR " familial atrial fibrillation" OR " atrial fibrillation, familial" ) |
| #2 | TITLE-ABS-KEY ( "cells, lymphoid" OR "lymphoid cells" OR "lymphoid cell" OR "cell, lymphoid" OR "lymphocyte" ) |
| #3 | ALL ( ratio ) |
| #4 | #2 AND #3 AND #1 |

Table S3. Summary of Adjustment Models and Main Covariates Across Included Studies.

| **Author** | **Country** | **Study Design** | **Outcome** | **NLR Cut-off** | **PLR Cut-off** | **SII Cut-off** | **Adjustment Status** | **Main Adjusted Covariates** |
| --- | --- | --- | --- | --- | --- | --- | --- | --- |
| Canpolat 2013 | Turkey | prospective | AF recurrence (late recurrence) | 3.15 | / | / | Adjusted (multivariable Cox regression) | Left atrial diameter, early AF recurrence, AF duration, nonparoxysmal AF type, age, high-sensitivity C-reactive protein |
| Ertaş 2013 | Turkey | retrospective | stroke | 3.17 | / | / | Adjusted (multivariable logistic regression) | Hypertension, hyperlipidemia, warfarin use |
| Im 2013 | Korea | retrospective | AF recurrence (early recurrence) | 5.60 | / | / | Adjusted (multivariable Cox regression) | Ablation time, left atrial volume, persistent AF type |
| [Guo 2014](https://pubmed.ncbi.nlm.nih.gov/?sort=pubdate&size=20&term=Guo+X&cauthor_id=24622430) | China | retrospective | AF recurrence (late recurrence) | 5.15 | / | / | Adjusted (multivariable Cox regression) | Left atrial diameter, body mass index |
| Saliba 2015 | Israel | retrospective | stroke | 3.00 | / | / | Adjusted (multivariable Cox regression) | Age, sex, and major cardiovascular comorbidities (CHA₂DS₂-VASc components, chronic kidney disease, chronic lung disease, anemia, malignancy) and medication use (aspirin, statins, β-blockers, ACE inhibitors/ARBs) |
| Yalcin 2015 | Turkey | retrospective | left atrial thrombosis | 2.59 | / | / | Adjusted (multivariable logistic regression) | Hypertension, diabetes mellitus, congestive heart failure, peripheral arterial disease, mean platelet volume, neutrophil percentage |
| Zhang 2017 | China | retrospective | AF recurrence (early recurrence) | 1.64 | / | / | Adjusted (binary logistic regression) | Left atrial volume index, AF duration, triglyceride, type of AF (non-PAF) |
| Fukuda 2018 | Japan | retrospective | left atrial thrombosis | 2.50 | / | / | Adjusted (multivariable logistic regression) | Diabetes mellitus, peak left atrial pressure |
| Li 2018 | China | prospective | left atrial thrombosis | NA | / | / | Adjusted (multivariable logistic regression) | D-dimer, B-type natriuretic peptide, left atrial diameter, E/Ea ratio |
| Bazoukis 2019 | Greece | retrospective | AF recurrence (late recurrence) | 3.90 | / | / | Adjusted (multivariable logistic regression) | Early arrhythmia recurrence, type of AF, procedure time |
| He 2021 | China | retrospective | left atrial thrombosis | 2.22 | / | / | Adjusted (multivariable logistic regression) | Left atrial diameter, history of heart failure, age, white blood cell count, CHA₂DS₂-VASc score, left ventricular ejection fraction, left ventricular end-systolic volume |
| Wu 2021 | China | retrospective | all-cause mortality | 1.67 | / | / | Adjusted (multivariable Cox regression) | Age, sex, body mass index, atrial fibrillation pattern, heart failure, cardiomyopathy, chronic obstructive pulmonary disease, chronic kidney disease, use of anticoagulants, ACE inhibitors, angiotensin receptor blockers, diuretics, left ventricular ejection fraction, hemoglobin, platelet count, high-sensitivity C-reactive protein |
| Etli 2022 | Turkey | retrospective | left atrial thrombosis | NA | / | / | Unadjusted | / |
| Tang 2022 | China | retrospective | left atrial thrombosis | 1.85 | / | / | Adjusted (multivariable logistic regression after age and sex adjustment) | CHA₂DS₂-VASc score, D-dimer, left atrial diameter |
| Xiang 2022 | China | retrospective | left atrial thrombosis | / | 160.90 | / | Adjusted (multivariable logistic regression) | Heart failure, NT-proBNP, eGFR, INR, left atrial diameter, left ventricular ejection fraction |
| Deng 2023 | China | retrospective | left atrial thrombosis | 2.57 | / | / | Adjusted (multivariable logistic regression) | Heart failure, previous stroke/TIA, left atrial diameter, ejection fraction, eGFR, WBC, CHADS₂ score, CHA₂DS₂-VASc score |
| Dolu 2023 | Turkey | retrospective | left atrial thrombosis | 2.63 | 131.50 | 693.60 | Adjusted (multivariable logistic regression) | WBC, left ventricular ejection fraction, spontaneous echo contrast |
| Fagundes 2023 | United States | prospective | all-cause mortality, stroke | / | NA | / | Adjusted (multivariable Cox proportional hazards regression) | Individual components of CHA₂DS₂-VASc score (congestive heart failure, hypertension, age, diabetes mellitus, stroke/TIA/systemic embolism, vascular disease, sex), HAS-BLED score components, Charlson Comorbidity Index, and race |
| Huang 2023 | China | retrospective | AF recurrence (late recurrence) | / | NA | / | Adjusted (multivariable logistic regression) | Left atrial diameter, ejection fraction, hypertension, diabetes mellitus, smoking, CHA₂DS₂-VASc score |
| Zhou 2023 | China | retrospective | left atrial thrombosis, all-cause mortality | 2.66 | / | 423.33 | Adjusted (multivariable logistic regression) | Age, gender, coronary heart disease, heart failure, hypertension, diabetes, stroke, oral anticoagulant use, left atrial diameter, left ventricular ejection fraction |
| Li 2024 | United States | retrospective | all-cause mortality | 8.37 | 159.77 | 1222.30 | Adjusted (multivariable Cox regression) | Age, Sex, Weight, CHF, Dementia, COPD, Renal disease, CABG, Cardiogenic shock, CCI, Aspirin, Beta-blocker, Amiodarone, Digitalis, Diuretics, Norepinephrine, Phenylephrine, Epinephrine, Statin, RR, Temperature, DBP, HR, Sodium, Glucose, Creatinine, Chloride, Calcium, PaO₂/FiO₂, pH, SAPS II |

AF, atrial fibrillation; ACE, angiotensin-converting enzyme; ARB, angiotensin receptor blocker; BMI, body mass index; BNP, B-type natriuretic peptide; CABG, coronary artery bypass grafting; CCI, Charlson Comorbidity Index; CHA₂DS₂-VASc, congestive heart failure, hypertension, age ≥75 years, diabetes mellitus, stroke/transient ischemic attack/systemic embolism, vascular disease, age 65–74 years, sex category; CHADS₂, congestive heart failure, hypertension, age ≥75 years, diabetes mellitus, stroke/transient ischemic attack; CHF, congestive heart failure; COPD, chronic obstructive pulmonary disease; DBP, diastolic blood pressure; D-dimer, fibrin degradation product test; eGFR, estimated glomerular filtration rate; E/Ea, ratio of early mitral inflow velocity to early diastolic mitral annular velocity; Hb, hemoglobin; HR, heart rate; INR, international normalized ratio; LA, left atrium/left atrial; LAVI, left atrial volume index; LV, left ventricle/left ventricular; LVEF, left ventricular ejection fraction; NA, not available; NLR, neutrophil-to-lymphocyte ratio; NT-proBNP, N-terminal pro–B-type natriuretic peptide; PaO₂/FiO₂, arterial oxygen partial pressure to fractional inspired oxygen ratio; PLR, platelet-to-lymphocyte ratio; RR, respiratory rate; SAPS II, Simplified Acute Physiology Score II; SII, systemic immune-inflammation index; TIA, transient ischemic attack; WBC, white blood cell count; /, not applicable.

**Table S4.** (A) The Newcastle-Ottawa Quality Assessment Scale Score for Cohort Studies.

| Study | Selection | | | | Comparability | | Outcome | | | Scores |
| --- | --- | --- | --- | --- | --- | --- | --- | --- | --- | --- |
|  | Representative-ness | Selection of  non-exposed | Ascertainment  of exposure | Outcome not present at start | Comparability on most important factors | Comparability on other risk factors | Assessment of outcome | Long enough follow-up (median≥1 year) | Adequacy  (completeness) of follow-up |  |
| Canpolat et al.2013 | * | * | * | * | * | - | * | * | * | 8 |
| Saliba et al.2015 | * | * | * | * | * | - | * | * | * | 8 |
| Wu et al.2021 | * | * | * | * | * | - | * | * | * | 8 |
| Fagundes et al.2023 | * | * | * | * | * | - | * | * | * | 8 |
| *indicates criterion met; - criterion not met. | | | | | | | | | |  |

**Table S4.** (B) The Newcastle-Ottawa Quality Assessment Scale Score for Case-Control Studies.

| Study | Selection | | | | Comparability | | Exposure | | | Scores |
| --- | --- | --- | --- | --- | --- | --- | --- | --- | --- | --- |
|  | Is the case definition adequate? | Representativeness of the cases | Selection of Controls | Definition of Controls | Comparability of cases and controls on the basis of the most important factor | Comparability of cases and controls on the basis of any additional factor | Ascertainment of exposure | Same method of ascertainment for cases and controls | Non-response rate |  |
|  |  |  |  |  |  |  |  |  |  |  |
| Ertaş et al. 2013 | * | * | * | * | * | - | * | * | * | 8 |
| Im et al. 2013 | * | * | * | * | * | - | * | * | * | 8 |
| Guo et al. 2014 | * | * | * | * | * | - | * | * | * | 8 |
| Yalcin et al. 2015 | * | * | * | * | * | - | * | * | * | 8 |
| Zhang et al. 2017 | * | * | * | * | * | - | * | * | * | 8 |
| Fukuda et al. 2018 | * | * | * | * | * | - | * | * | * | 8 |
| Li et al. 2018 | * | * | * | * | * | - | * | * | - | 7 |
| Bazoukis et al. 2019 | * | * | * | * | * | - | * | * | * | 8 |
| He et al. 2021 | * | * | * | * | * | - | * | * | * | 8 |
| Etli et al. 2022 | * | * | * | * | * | - | * | * | * | 8 |
| Liang et al. 2022 | * | * | * | * | * | - | * | * | * | 8 |
| Xiang et al. 2022 | * | * | * | * | * | - | * | * | * | 8 |
| Deng et al. 2023 | * | * | * | * | * | - | * | * | * | 8 |
| Huang et al. 2023 | * | * | * | * | * | - | * | * | * | 8 |
| Zhou et al. 2023 | * | * | * | * | * | - | * | * | * | 8 |
| Li et al. 2024 | * | * | * | * | * | - | * | * | * | 8 |

*indicates criterion met; - criterion not met.


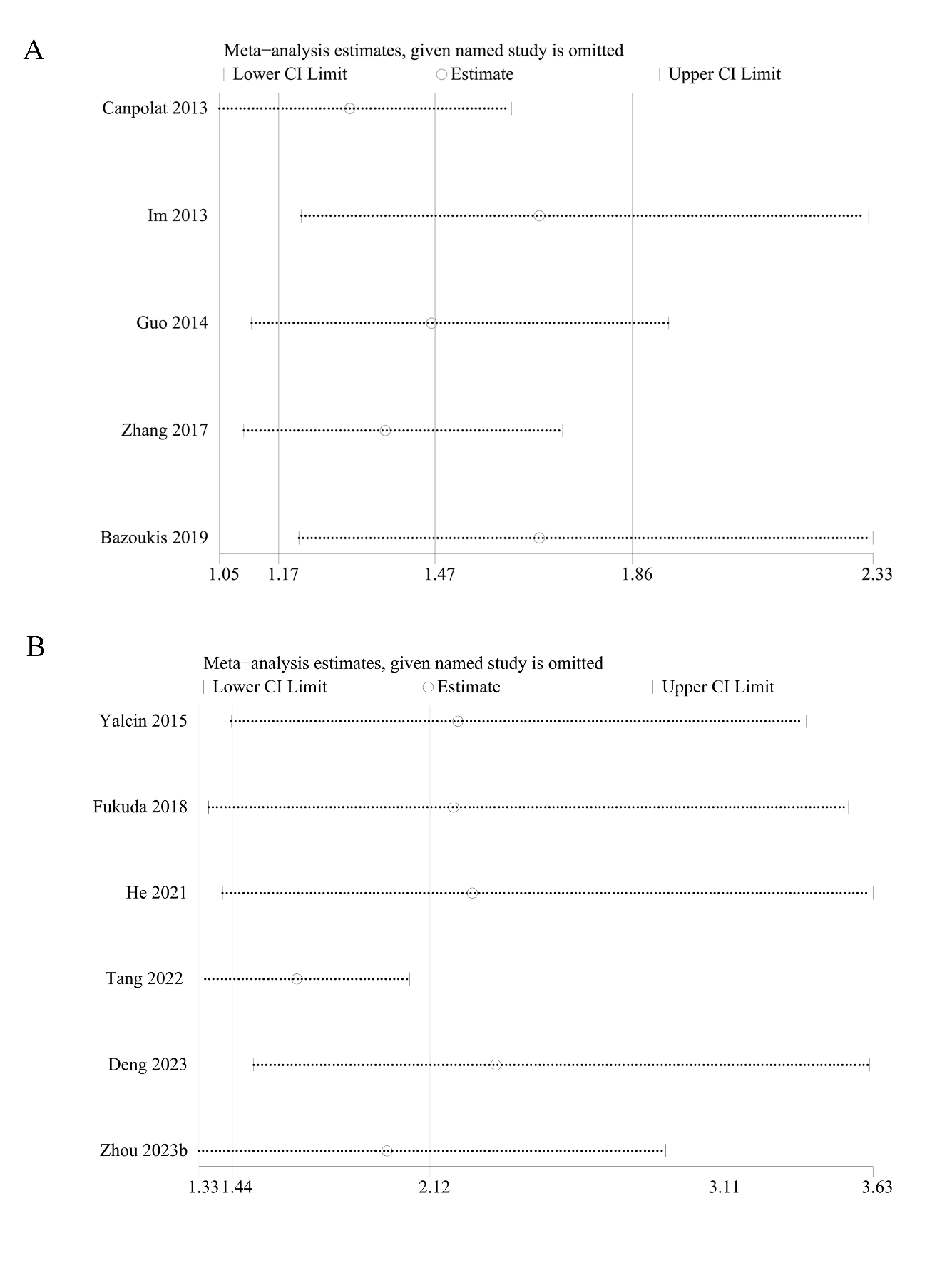


**Figure S1.** Leave-one-out sensitivity analyses of ORs for associations between NLR and different clinical outcomes in patients with atrial fibrillation: (A) AF recurrence; (B) Left atrial thrombosis. The x-axis represents the pooled effect size (OR) with 95% CI, and the y-axis lists the individual studies sequentially omitted.


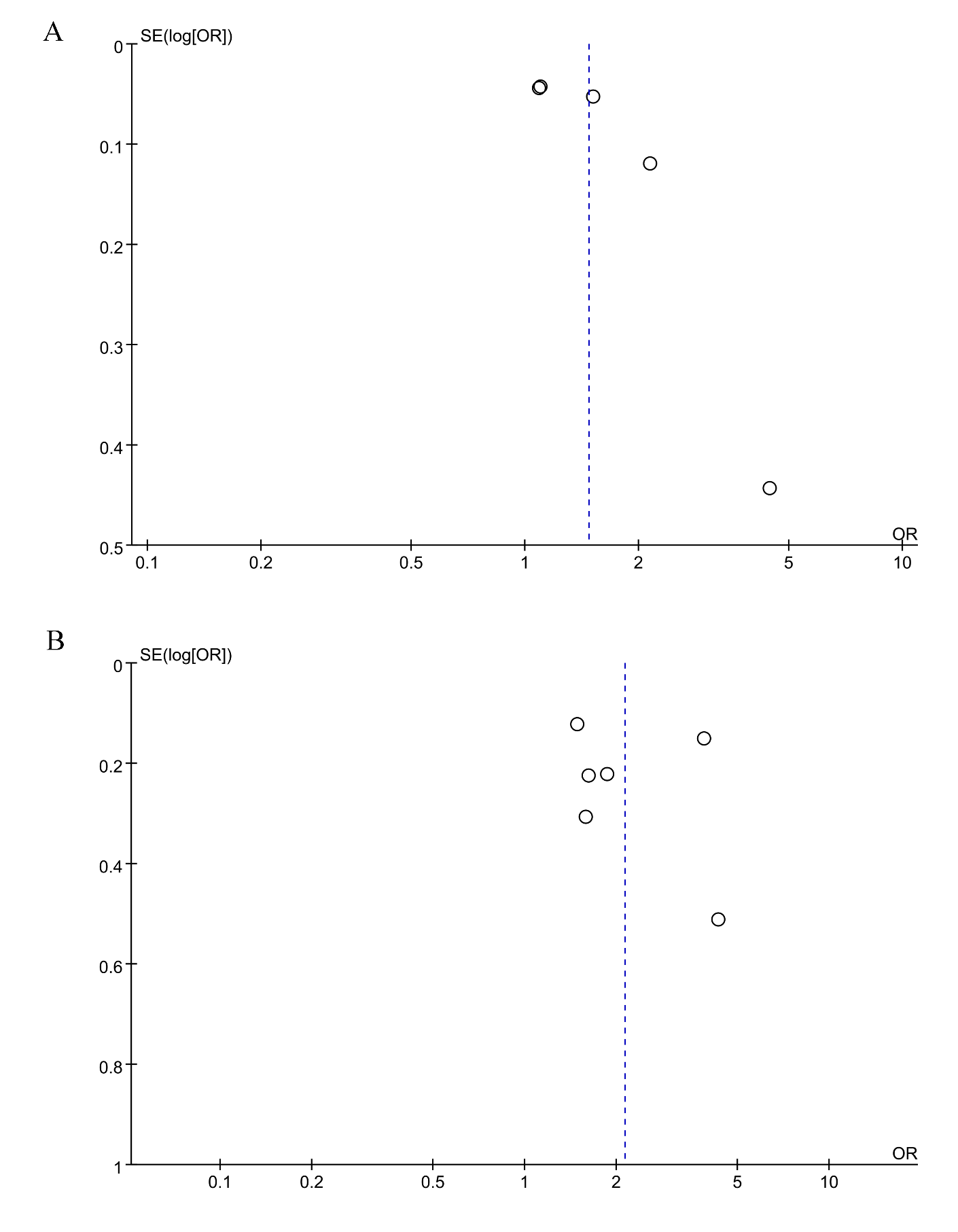


**Figure S2.** Funnel Plots of ORs for Associations Between NLR and Different Clinical Outcomes in Patients with AF: (A) AF recurrence; (B) Left atrial thrombosis. The x-axis represents the effect size (OR), and the y-axis represents the standard error of the log-transformed estimates.
